# Using the Consolidated Framework for Implementation Research to examine implementation determinants of specialty mental health probation

**DOI:** 10.1186/s40352-019-0098-5

**Published:** 2019-12-05

**Authors:** Tonya B. Van Deinse, Alicia Bunger, Stacey Burgin, Amy Blank Wilson, Gary S. Cuddeback

**Affiliations:** 1 School of Social Work, University of North Carolina at Chapel Hill, 325 Pittsboro Street, CB#3550, Chapel Hill, NC 27599 USA; 2 College of Social Work, The Ohio State University, 1947 N. College Rd., Columbus, OH 43210 USA; 3 Department of Psychiatry, University of North Carolina at Chapel Hill, 101 Manning Dr #1, Chapel Hill, NC 27514 USA

**Keywords:** Probation, Implementation, Mental illness, Consolidated framework for implementation research

## Abstract

**Abstract:**

**Background:**

Specialty mental health probation (SMHP) is designed to improve outcomes for the large number of people with serious mental illnesses who are on probation and/or parole. The evidence for specialty mental health probation is promising; however, little is known about the implementation challenges and facilitators associated with SMHP. To address this gap, we used the consolidated framework for implementation research (CFIR) to analyze 26 interviews with stakeholders representing multiple agencies involved in the implementation of SMHP.

**Results:**

Results indicate a number of challenges and facilitators related to the inner setting, outer setting, implementation process, and characteristics of individuals.

**Conclusions:**

Findings suggest that complex and cross-sectoral interventions are context-dependent and introduce a number of challenges and facilitators related to multiple CFIR domains. Consequently, agency administrators implementing these types of interventions should consider small pilot studies and develop implementation strategies tailored to the local implementation context.

## Background

Nearly 7 million adults in the United States are under correctional supervision and almost 70% of these individuals are on probation and/or parole (Kaeble and Cowhig 2018). Although the census for community supervision has gradually declined since 2007 (Kaeble and Cowhig 2018), the prevalence of mental illnesses among adults on probation remains high with estimates ranging between 16%–27% of the approximately 4.65 million probationers and parolees in the United States (Crilly et al. 2009; Ditton 1999; Kaeble and Cowhig 2018; Van Deinse et al. 2018). When considered in the context of inadequate mental health training for probation officers and high workload demands, as well as higher rates of infractions and violations among adults with mental illnesses on probation, the high prevalence rates of mental illnesses among probationers pose significant challenges for state and local probation agencies (Eno Louden and Skeem 2011; Porporino and Motiuk 1995; Skeem and Eno Louden 2006; Van Deinse et al. 2017). Criminal justice authorities need effective interventions to address the public health and public safety challenges presented by adults with mental illnesses on probation.

In 2002, the Council of State Governments (CSG) encouraged specialized approaches for community supervision of individuals with mental illnesses, including reduced caseloads, mental health training for officers, and assignment to designated mental health caseloads for the duration of their supervision (Council of State Governments 2002). Since CSG’s endorsement, a number of studies have examined such specialized approaches (e.g., Manchak et al. 2014; Skeem et al. 2006, 2017; Wolff et al. 2014) and at last count, specialty mental health probation (SMHP) had been implemented in nearly 140 probation agencies across the United States (Skeem et al. 2006). SMHP is a complex, transdisciplinary, and multi-component intervention that has emerged as one strategy to supervise individuals with mental illnesses who are on probation. Although the structure and implementation of SMHP vary by criminal justice agency, five key elements are identified as the prototypical model: (a) probation caseloads consisting exclusively of individuals with mental illnesses; (b) reduced caseload size; (c) ongoing mental health training for officers; (d) a problem-solving supervision orientation; and (e) collaboration with internal and external resources to link probationers with services and supports (Skeem et al. 2006). Although there are no published studies of randomized controlled trials of SMHP, results from quasi-experimental studies indicate SMHP’s potential to improve mental health and criminal justice outcomes (Manchak et al. 2014; Skeem et al. 2017; Wolff et al. 2014).

Despite its widespread use, there is little focus on the implementation of SMHP (Manchak et al. 2014), specifically, or on interventions within adult community corrections (Alexander 2011; Gendreau et al. 1999; Taxman and Belenko 2011), in general. This lack of focus on implementation underscores two critical and related limitations in the research on SMHP that impede efforts to build its evidence base. First, there is no published research on the determinants that impact implementation of SMHP. Determinants are multi-level contextual factors – such as individual self-efficacy and knowledge, organizational readiness, external policy context, etc. – that can inhibit or enable intervention implementation outcomes, such as adoption and fidelity (Damschroder et al. 2009; Nilsen 2015).

Second, SMHP is intended to link mental health and correctional systems and relies on collaboration across an expansive array of stakeholders, including probation officers, probation administrators, and mental health service providers. As a result, contextual features of both systems influence implementation and service delivery. Stakeholders in both corrections and mental health have unique perspectives based on their implementation and service provision responsibilities, all of which are necessary for a comprehensive understanding of SMHP implementation. Consequently, lack of knowledge about key determinants that impact implementation can result in wide variation in model implementation which impacts internal validity and the generalizability of research on SMHP.

Addressing these gaps is an essential first step and will facilitate the development and testing of implementation strategies. An implementation strategy is “a systematic intervention process to adopt and integrate evidence-based health innovations into usual care” (Powell et al. 2012, pg. 2). Implementation strategies can enhance the uptake of an intervention by leveraging facilitators (i.e., determinants that enhance implementation) to address barriers (i.e., determinants that inhibit implementation; Colquhoun et al. 2017). Given the well-documented impact of multi-level factors (e.g., characteristics of individuals, external policy context, organizational readiness, leadership) on successful implementation of interventions (Aarons et al. 2011; Damschroder et al. 2009; Nilsen 2015) and the complexity and transdisciplinary nature of SMHP (i.e., a criminal justice intervention that requires collaboration and coordination across external systems), development of implementation strategies to enhance adoption of SMHP’s five core components is essential. Understanding the determinants that impact implementation of SMHP and how these factors may vary by setting (urban vs. rural counties; local vs. statewide position) is the first step in developing implementation strategies (Colquhoun et al. 2017) to enhance adoption and dissemination of SMHP.

To address these limitations in the implementation research on SMHP, this study examines the determinants that impact implementation of SMHP in one rural and one urban county using the Consolidated Framework for Implementation Research (CFIR; Damschroder et al. 2009). CFIR is a widely used framework that describes a multi-level set of constructs to enhance implementation and dissemination (Damschroder et al. 2009; Nilsen 2015).

## Methods

### Study context

In 2011, the state’s Department of Public Safety (DPS) convened a meeting with stakeholders from the Department of Health and Human Services (DHHS) and Treatment Accountability for Safer Communities (TASC) to discuss implementation of SMHP. DPS also engaged a research team from the University of North Carolina at Chapel Hill to develop a pilot study of SMHP in two counties – one rural and one urban county – in which individuals with serious mental illnesses on probation would be randomly assigned to either standard probation or SMHP. Representatives from DPS, DHHS, and TASC formed the Executive Committee for SMHP and members of the research team formed the Implementation Team. Partners and stakeholders at the local level included DPS and TASC representatives, as well as representatives from the local managed care organizations (MCO) that manage local mental health and substance use services.

The core components of the SMHP pilot were based on the prototypical SMHP model advanced by Skeem et al. (2006) and included the following: (a) probation caseloads consisting exclusively of individuals with mental illnesses; (b) reduced caseload size; (c) ongoing mental health training for officers; (d) a problem-solving supervision orientation; and (e) collaboration with internal and external resources to link probationers with services and supports. In terms of implementation of the SMHP model, DPS was responsible for establishing policies and procedures to create designated mental health caseloads and to reduce SMHP officers’ caseload size. Stakeholders from TASC and the MCOs helped facilitate contact with local mental health and substance use service providers. The Implementation Team was responsible for coordinating and providing initial and on-going mental health training and clinical case consultation for the SMHP officers and, in concert with DPS, helping the SMHP officers develop a problem-solving supervision orientation that balanced public safety and the behavioral health needs of the individuals on the specialty mental health caseloads. Further, the Implementation Team coordinated and facilitated stakeholder engagement meetings that were designed to educate community partners about the pilot and to build relationships between partnering agencies.

### Study design

This study reports on the results of the implementation arm of a hybrid effectiveness-implementation study (Curran et al. 2012) that examined trends in participant responses regarding the determinants of the implementation of SMHP in one rural and one urban county in a southeastern state. The implementation arm of the study was a formative evaluation that occurred during early implementation of SMHP. Specifically, researchers conducted semi-structured interviews with key stakeholders who were involved in the planning and implementation phases of the study. These interviews were conducted after study enrollment began and before all core components of the model were fully implemented. This study was approved by the Institutional Review Board at the University of North Carolina at Chapel Hill.

### Participants

Participants included 26 individuals, who were selected and recruited to participate in the study using a purposive sampling approach. Individuals were selected if they were part of the SMHP implementation process and represented one of the key SMHP stakeholder groups: the state and local levels of DPS and TASC; state representatives from DHHS; local representatives from the managed care organizations for mental health and substance use services; and representatives from the university research partner who served as the Implementation Team.

Of the 26 respondents, 54% (*n* = 14) were female, 85% (*n* = 22) were White/Caucasian, 15% (*n* = 4) were African American. In addition, 19% (*n* = 5) were statewide representatives of DPS, TASC, and DHHS; 65% (*n* = 17) were local representatives from probation, mental health and TASC; and the remaining 15% (*n* = 4) were members of the Implementation Team from the university research partner which liaised between state and local entities during the implementation process. Lastly, 46% (*n* = 12) of the respondents represented the criminal justice system, 23% (*n* = 6) represented the mental health system (i.e., DHHS and local MCO), and 15% (*n* = 4) were local TASC representatives who served as a bridge between probation and the mental health and substance use services systems.

### Data collection

Researchers developed a 17-question semi-structured interview guide focused on participant demographic and background information, role in SMHP implementation, perceived challenges and facilitators during the SMHP planning and implementation stages, expected long-term outcomes of SMHP, and perspectives on mental health training approaches for SMHP officers. For the purposes of this study, researchers focused on questions related to the challenges and facilitators of SMHP planning and implementation. Of the 26 interviews, 24 were conducted in person and two interviews were conducted over the phone. Interviews ranged in length from 25 to 75 min and all interviews were audio-recorded and transcribed verbatim.

### Data analysis

Data analysis for this exploratory study occurred in two steps. In the first step, a general inductive approach (Thomas 2006) was used to develop categories from the raw data, namely text in which participants described a challenge or facilitator to implementing SMHP. During this process, two researchers independently coded transcripts and used consensual qualitative research methods (CQR; Hill et al. 2005) in which both researchers compared codes and discussed coding inconsistencies until full agreement was reached. A third researcher with extensive experience in qualitative research methods audited all stages of the analysis process.

In the second step of the analysis, researchers used a deductive coding strategy wherein the implementation challenges and facilitators that were identified during the general inductive coding process were categorized using CFIR. CFIR consists of five domains – innovation characteristics, outer setting, inner setting, characteristics of individuals, and implementation processes – each of which has a subset of constructs derived from theories for effective implementation (Damschroder et al. 2009). The research team used CQR methods again to resolve coding discrepancies.

### SMHP and the consolidated framework for implementation research

In this study, CFIR was chosen as a deductive coding strategy for SMHP implementation barriers and facilitators for several reasons. First, CFIR is a widely recognized determinant framework (Nilsen 2015) that includes a comprehensive and multi-level set of constructs that help to explain and predict implementation (Damschroder et al. 2009). Second, resources pertaining to CFIR domains, templates for gathering data and other tools were readily available online (see www.cfirguide.org) to assist with multiple aspects of the study, most notably the coding process. Third, given the dearth of implementation research within the correctional research literature, the research team wanted to connect the implementation of SMHP to existing theoretical and empirical literature within implementation science.

There are five broad domains within CFIR: (a) innovation characteristics; (b) outer setting; (c) inner setting; (d) characteristics of individuals; and (e) implementation processes (Damschroder et al. 2009). Within the CFIR framework, *innovation characteristics* refer to characteristics associated with the intervention including the perceived quality of the intervention, its complexity, how it is designed, and its cost. For this study, innovation characteristics are those that are associated with the five core components of SMHP: reduced caseload size, problem-solving orientation, exclusive mental health caseload, ongoing training, and interfacing with external organizations (Skeem et al. 2006).

The *outer setting* refers to the environment in which the agency exists, including “the economic, political, and social context within which an organization resides” (Damschroder et al. 2009, p.5). Here, the outer setting refers to the counties in which SMHP was implemented as well as the partnering organizations (e.g., mental health, TASC, etc.). The *inner setting* domain refers to the characteristics (e.g., agency communication, culture, climate, and leadership) associated with the organization implementing the innovation. The inner setting refers to the characteristics associated with the local probation agency or the larger DPS entity.

*Characteristics of individuals* refers to the characteristics (e.g., individuals’ knowledge about the intervention and their sense of self-efficacy) of those involved in the intervention. In this study, individuals included the officers who were delivering the intervention as well as internal and external partners associated with the pilot, including implementation team members. Lastly, *implementation process* refers to the characteristics of the strategies and processes (e.g., planning, engagement, executing, and evaluating) used to facilitate the implementation of the intervention. For the purposes of this study, the implementation process refers to tactics used to enhance the implementation of SMHP at the county or state level. Strategies and tactics used during implementation are not features of the intervention itself.

## Results

The results of the study are organized by number of respondents endorsing implementation determinants in the five CFIR domains. Additional information about specific constructs within the CFIR domains is provided to illustrate the application of the domain to the context of SMHP implementation.

### Implementation challenges of SMHP

Participants identified a variety of SMHP implementation challenges related to three of the five CFIR domains: the inner organizational setting, the nature of the implementation process, and the outer setting.

#### Inner setting

Nearly all participants (96.15%, *n* = 25) described implementation challenges associated with the inner organizational setting in probation agencies. Specifically, participants discussed *compatibility*, or the fit between SMHP and the values, norms, and perceptions of the probation agency and existing workflows (Damschroder et al. 2009). During interviews, compatibility issues emerged as participants discussed how the time-intensive nature of the SMHP model was, at times, incompatible with officers’ heavy and varied work demands, and was therefore perceived as an extra “burden.” For example, one SMHP officer explained:

Well, the first thing that comes to my mind in terms of challenges has been time. Because you know, you have a full caseload of defendants, not just focusing on individuals that have mental health disorders. I’m not just focusing on the training. I’m not just focusing on implementing what I have learned, or how we are handling this pilot program. So it is fitting everything into the schedule in the time frame that I have. So I would say just trying to meet the needs that are there and learning what I needed to do and doing it. Time, basically (Participant 015).

This perspective was consistent across stakeholders in the study. For instance, a participant from a mental health partner commented:

I feel like, well of course challenges with any probation officer, juvenile or adult, is workload. They’re so busy doing all these other things. I feel like with [SMHP] they should have had their plate wiped clean and then given them this pilot to solely focus on. I feel like they were kind of thrown into it, with already this caseload and then an extra burden on top of training … court and all these other things (Participant 024).

In addition, officers must balance other workload demands including intensive trainings:

Another interesting issue is that these officers already have to go to so many trainings because they have all their law enforcement trainings. [SMHP officer] told me the other day that [they are] going to a [week-long training]. So that’s a week that [they are] away from cases. So they have a lot of training … they have to have their routine CPR and firearms training … and they’re going to have to do so many mental health trainings (Participant 005).

Another officer explained: “I told [my supervisor], I said look, I got 80 cases. I’m trying to do the [SMHP pilot]...it’s too much and [my supervisor] agreed. This pilot cannot be done with that amount of cases” (Participant 013). The challenge of large caseload sizes was also noted by a member of the implementation team who stated: “They have huge caseloads, a lot of paper work to do, a lot of visits, and a lot of other stuff, and I think the perception is that this is just one more thing added on to my already stressful job, so there’s that” (Participant 023). These comments indicate that the components of SMHP were incompatible with the existing work demands of officers (e.g., high caseload sizes, trainings), which made it difficult to implement the model as intended.

Reducing caseloads was challenging because of limited staff *resources*, which is an indicator of an organization’s readiness for implementation and is characterized by the presence of organizational resources that are committed to the implementation of the intervention (Damschroder et al. 2009). In the context of this study, available resources of the inner setting can refer to staffing patterns, probation officer turnover, physical space, and dedicated time. To ensure probation officers have sufficient time, the SMHP model requires caseload reductions (e.g., from 70 to 40) for specialty mental health officers (Skeem et al. 2006). Yet staff vacancies and new hires were an impediment to reducing caseload sizes during the initial implementation of SMHP. One respondent explained,

I mean we must have about 20 new officers. So that is kind of challenging too. We have a very young staff, staff that are coming right off the street and don’t have a lot experience. We don’t have a lot of veteran staff. So that’s kind of a barrier to this too. The [SMHP] officers are overwhelmed with cases because the new staff who haven’t gone to basic yet can’t get cases. Caseload numbers are not drastically too high, we’re not in crisis, but they are high (Participant 012).

Another participant from probation explained additional challenges related to caseloads that may help understand the difficulty with caseload reduction.

… when I’m looking at the [number of cases] and keeping caseloads balanced, it’s keeping our [SMHP officers], keeping their numbers balanced with everyone else’s now until a decision is made to reduce their caseload if that ever comes about. It’s keeping their numbers balanced with everyone else (Participant 011).

This quote suggests that there are some complicating factors that impact caseload reduction, such as the need to balance cases across SMHP officers and standard officers and that a decision about caseload reduction had not yet been made or communicated.

#### Implementation process

Two-thirds (65.38%, *n* = 17) of the total sample described challenges with the implementation process – i.e., strategies and tactics aimed at facilitating the implementation of SMHP (Damschroder et al. 2009). The primary implementation process challenge was related to collaboration across different stakeholder systems (e.g., probation, mental health, managed care), including engagement, communication (i.e., different terminology and language across disciplines), and role confusion among the probation officers, mental health clinicians, and TASC providers.

Many of the challenges related to the implementation process were related to stakeholder engagement, which refers to the ways in which individuals were involved in the implementation process. For instance, Executive Committee members and Implementation Team members reflected on communication challenges with respect to bridging mental health and criminal justice terminology and language. One respondent (Participant 003) described this challenge as “bridging the gap between academics and operations” and an Executive Committee member described the challenge of engaging across systems in the following way:

With me, it was the language and understanding all the different terms and then how to put it in layman’s terms, and then how to relate it to the probation officers. ‘Cause I’m still getting a lot of that … “this is too medical, this isn’t our role,” but how do we put it in terms that, “it’s not your role to be the counselor, but it is your role to understand the terms and understand the process to get the person to services.” And then the challenges of you know, working with TASC, and ourselves, and then with [the Implementation Team], we just all speak different languages and kind of, kind of understanding what the priorities are and you know at times I think I was frustrated, but that was because I was still learning myself (Participant 009).

In addition, an Implementation Team member commented:

I would say that the biggest thing I think was really language and I think there were times in meetings where it seemed we were on different pages talking about different things maybe in a completely different book but when in reality we really weren’t. I think getting the language down, getting the acronyms down, learning how to speak probation, how to speak mental health, and all of the stuff that comes with this – the different policies, different procedures, understanding each other’s systems – I think that was a challenge. I think everyone did very well with that challenge and recognizing that this is really just an issue of not totally understanding everyone else’s environment and language. I’d say that was the biggest one (Participant 023).

These statements describe the challenge of beginning to engage in discussions around the planning and implementation of SMHP due to differences in how mental illness is described and talked about. For instance, there were inconsistencies in the use and understanding of terms like “dual disorder,” “co-occurring,” and “case management.” One respondent from probation noted this discrepancy in language:

I would say that a challenge might have been the vocabulary and making sure that what we were saying meant the same thing, so that the terms we were using – we use case management in one sense which means supervision, I think y’all use case management for more of that medical, making sure that the person is doing what they’re supposed to – so we need to make sure that vocabulary-wise what we were saying was the same thing (Participant 002).

Participants also described role confusion and difficulty identifying one another’s distinct agency responsibilities for implementing and delivering SMHP. One participant described the challenge of working across systems in the following way:

Probably role confusion or not knowing different roles. So here’s TASC and what are they going to bring to the table? Here’s DPS and what are they going to do? Here’s [the university]. And at those larger meetings, we had a lot of folks and folks that we couldn’t even really identify from our perspective, you know, who was TASC and who was DPS and who was DHHS? So just knowing all the players and different players moving in and out. I think it was a size issue, being just a big group with lots of perspectives and opinions. I think ironing out the details – what we were going to do, who were we going to identify, who were we going to target – it took some education on our part (Participant 004).

In addition, a respondent from probation commented:

So everybody … there’s a clear understanding of what your role is and our role is and when we’re supposed to go, maybe, slightly beyond our traditional role and you’re comfortable with that. I think that’s a lot. Because there were a couple situations where I think TASC was like, “Oh, no – you don’t do that. That’s our job. So, you know, if you need that done, you need to call us, first.” And so having that understanding of the process. This is what DPS does and this is how they do it. This is TASC’s role (Participant 003).

#### Outer setting

Half (50%, *n* = 13) of respondents indicated implementation challenges as a result of issues in the external system and policy (outer) setting. Participants described how limited availability of community resources influenced SMHP implementation. For instance, one SMHP implementation challenge related to availability of resources was transportation. Multiple respondents from the rural county identified transportation as a key challenge. For example one respondent said, “… as far as barriers, [this rural county] is a very large county. It’s debatable whether it’s the largest in the state or second largest, but it’s a very large county, a very poor, very rural county. So transportation can be an issue” (Participant 011). Another participant explained:

One of the biggest factors the rural county faces is the transportation issue. And I really don’t know what can be done about that from [the Implementation Team]. I’m always going to throw out transportation most of the time in anything that we’re talking about as far as what our agency participates in, what our people – what our offenders do. In a rural county, that is – you’ve got to understand – it is a really big issue, especially when we don’t have the public transportation like a lot of urban places do. Some counties have county-funded transportation. Here, it’s very limited and they even suspended it because of budget constraints – what little bit they do have here. I don’t know what you could do as far as this project goes moving forward looking into transportation, but I do consider transportation to be a big issue in a rural county (Participant 019).

In addition, another rural participant responded:

Probably one of the biggest challenges is transportation, I would think. We have a little bus situation but lots of times I think it’s based on Medicare or whatever funding they can get. That’s another issue. People can’t get to their appointments so then they have to pay somebody … an exorbitant amount of money to drive them from one part of the county to another part of the county. And gas money … That’s one the biggest things, that’s one of the biggest challenges here is transportation (Participant 020).

In addition to transportation, respondents also indicated implementation challenges that were related to the structure and delivery of mental health services within the context of changes that occurred to the mental health services system. For instance, participants noted that prior to the pilot study mental health service delivery changed from a more centralized model where services could be accessed at one location to a more dispersed model with many service providers (whose services and operating status fluctuated). Under this new structure, participants perceived that mental health resource availability was unstable:

Well, when we did have the mental health agencies in each county, it was easy. Just make a referral to mental health, go out to the county complex, see someone there. But when they did away with mental health, that’s when, in my opinion, it became more difficult for us as officers. Because that was a one stop shop – you sent them there [and] they’ll know what you need, they’ll get you the help, point you in the right direction. But once that went away, we knew that was gone, but we didn’t … we were never told that now that mental health is gone, this is the agency that you go to now. It seemed like everyone and their brother was hanging a shield. We were having people coming by all the time. “Here’s my card. We’re open now. We can do this, we can do that.” Then we tell the [probationers] and start sending them and then two weeks later, the shield [is] gone and [the mental health service providers] are gone. They left town. Then you would ask the offender, have you been to see your provider? Have you been to see your counselor? “Yeah, but when I got there the door was locked.” So that posed a problem (Participant 011).

Although the challenges identified here could apply to individuals on probation who have mental illnesses in general – including those not enrolled in the pilot study – this lack of available mental health services may be particularly challenging for SMHP officers since a key component of SMHP is interfacing with external resources. In fact, one SMHP officer noted “there aren’t as many mental health [service] providers as there were [at] one time … Not having the resources – that definitely would be the biggest barrier. Not having the resources to rely on to assist the individual in a proper manner” (Participant 015). The CFIR model does include this same construct (i.e., availability of resources) within the inner setting domain; however, that construct does not account for the presence or absence of available resources (e.g., transportation, mental health services, other community services) in the community, which participants indicated were important implementation challenges.

### Implementation facilitators of SMHP

Participants identified a number of factors that facilitated the implementation of SMHP. These factors were primarily related to characteristics of individuals, the implementation process, and the inner and outer setting.

#### Characteristics of individuals

Of the 26 stakeholders, 88.46% (*n* = 23) indicated that characteristics of individuals facilitated the implementation process. Participants described how characteristics of individuals that may have had a positive impact on the implementation of SMHP. For instance, participants noted that officer expertise and relationships with others allowed them to help colleagues with difficult cases:

Those officers there are also very interactive and they talk to their fellow officers, I think, about the project and explain it to them. And that’s really how we get a lot more referrals directly from officers in Sampson, because I think that those officers have a trouble or troubling case and they go to the specialty officers, and the specialty officers recommend that they refer them to the study so that they can take part in that (Participant 022).

Respondents also noted the importance of officers to be willing to think beyond traditional approaches and “step outside”:

Learning more about the population and better ways to communicate with this population, as well as a willingness to step outside of their offices and go into a provider’s office to get to providers, since providers aren’t necessarily in a place where they can step out of their world into probation world because I think they’re more restricted. So I think that having that willingness of the officers, and ability to meet providers is also important and shows buy in (Participant 022).

One respondent suggested that an officer’s years of service were an asset to SMHP and mental health supervision:

I really think that veteran officer can lead … can really help with the buy-in of the project. I just feel like that’s important and the veteran officer is somebody that the newer officers can look to and go to for questions or get experience … how to handle this or how to handle that, what’s been your experiences with this (Participant 019).

#### Implementation process

Although respondents indicated challenges associated with the implementation process, they also noted a number of facilitators as well. For instance, 80.77% (*n* = 21) of the 26 stakeholders indicated that tactics related to the implementation process were helpful. Although engaging stakeholders across systems was challenging (as noted in the previous section), respondents also believed that this process helped facilitate the implementation of SMHP. For instance, in terms of CFIR constructs, respondents indicated that engagement with the *formally appointed implementation leaders* was a facilitator. Having formally appointed implementation leaders –the Implementation Team – that assisted with coordination, communication, coaching and troubleshooting throughout the planning process was an important asset that help manage implementation tasks. One participant explained:

Well, yeah, having [the Implementation Team] to run things by and having the positive feedback from [them] has been really great. The encouragement that we have received when we may feel, “oh goodness I don’t know what I’m doing” or “I don’t know how to handle this,” it seems as if it’s been laid out, you know, like a map as far as I’m concerned. So, having that available and having it be available to refer to has made it [a] very positive and rewarding experience (Participant 015).

Another participant recalled:

I remember thinking this was going to be too huge. How in the world are we going to do all of this and it’s that bite the elephant one bite at a time kind of thing. So [the Implementation Team] really helped break it down, did a tremendous amount of the work for us, and just kind of kept us on track (Participant 002).

Further, another respondent commented:

Well, the work that [the Implementation Team] did in keeping everything coordinated certainly was helpful in getting the emails, having someone follow-up after each meeting with emails that kind of keep it in your mind – okay – that this is what we said we were going to do, and this is what we’ve got to have done by the next time. Just having someone to coordinate that process is great. DPS’s willingness to help – to provide the meeting space – all of that was helpful (Participant 021).

Responses indicated that the engagement of the Implementation Team provided necessary follow through, logistical assistance, timely feedback, and general support, and assisted the Executive Committee with coordination, follow up and project management.

#### Outer setting

Although fewer participants (46.15%, *n* = 12) indicated that characteristics related to the outer setting were helpful in implementing SMHP, given the dependence of the SMHP model on factors related to the outer setting, it is worth noting some of the relevant constructs indicated by participants. For instance, a key component of SMHP requires greater interface with external organizations and one of the key facilitators associated with the outer setting pertained to *cosmopolitanism,* “the degree to which an organization is networked with other external organizations” (Damschroder et al. 2009, p. 7). Given the role of SMHP officers in service connection, having network connections would enhance implementation of the model. For instance, one SMHP officer described their new connections with local mental health providers saying: “And the agencies, the mental health agencies that I’ve formed a very positive rapport to be able to call and ask questions or sending information. We work really well together” (Participant 015). Another SMHP officer noted:

That’s the other thing, you’ve [asked] about what’s been [helpful], I mean just the relationship with [the MCO] has been extremely [helpful]. I mean people have had questions and I’ve been able to give them [my contact’s] phone number and say here, call [my contact] and he’ll help you. If he can’t help you right this second, he’ll get right back to you (Participant 014).

In addition, other partners at the local level discussed how these new contacts within the community had already helped SMHP officers to implement boundary-spanning tasks associated with SMHP. For example, a local partner further described SMHP officers’ new contacts with mental health agencies:

Every time we go to [an agency], [the SMHP officer] gives feedback to that provider about how we met with the previous providers and it went really well, and how [the officers] have found it really useful because [they know] if [they] has an issue [they] know who [they] can pick up and call. If [the mental health provider] knows who [the officer] is they’re going to respond immediately because we’ve been out there and established those relationships (Participant 024).

Creating these relationships across networks is a key facilitator given the role of officers within the SMHP model and the need for SMHP officers to engage and collaborate with external resources to access the treatment needs of the probationers on their caseloads.

#### Inner setting

Although the inner setting domain did not emerge as a key facilitator (in aggregate, only 30.77%, *n* = 8 of the 26 stakeholders indicated facilitators related to the inner setting), the wide variation in stakeholder perspectives is worth examining. Specifically, although no respondents from the mental health or TASC partners indicated facilitators related to the inner setting, all members of the Implementation Team and more than a third of probation representatives indicated facilitative factors of the inner setting. This variation across respondents may be reflective of the knowledge respondents had about factors and events promoting implementation within the probation agency, consequently, salient factors noted by those most familiar with the inner setting are worth noting. For instance, an identified facilitator within the inner setting pertained to leadership engagement within the CFIR construct *readiness for implementation* (Damschroder et al. 2009)*.* The CFIR construct *leadership engagement* refers to the leadership’s level of involvement and commitment to SMHP. Respondents described leadership as being open and committed to the innovation and actively promoting the implementation within the DPS setting. For instance, one respondent said “I mean, from the beginning, [the Director] and the Commissioner have been very supportive – it was something they wanted, it was something they felt was very important” (Participant 003). Another respondent also commented on the Community Corrections Director’s involvement in the pilot saying, “Well, being present I think was really helpful, just the fact that she was there and then requiring other people’s presence at the meetings that we had and at every step of the way she communicates how important the project is” (Participant 023). Another respondent noted that an implementation facilitator was “certainly the buy-in with the state at the top. I mean, if you think about it, that’s really the buy-in we need. That certainly was a big help” (Participant 004).

## Discussion

To address limitations in our understanding of the implementation of SMHP, we conducted a hybrid effectiveness-implementation study using qualitative methods and semi-structured interviews. Results indicated a number of implementation challenges related to multiple CFIR domains (e.g., inner setting implementation process, outer setting). Specifically, participants noted that the demands of the existing workload of the SMHP officers made it challenging to implement components of the SMHP model (e.g., increased connection and networking with external providers). In addition, participants indicated challenges related to working across the criminal justice and mental health systems. Participants noted differences in, and confusion about, language and terminology used across partners (e.g., challenge of understanding clinical terminology among criminal justice partners) and the potential for role confusion when probation officers are collaborating and coordinating with mental health service partners. Participants also noted how challenges related to the external local environment – e.g., connecting with available resources in the community – impacted implementation of SMHP.

Study participants also noted a number of factors that facilitated the implementation of SMHP. First, participants indicated that characteristics of the probation officers seemed to help with SMHP implementation. For example, officers were described by their expertise, knowledge of the service system, and their willingness to “step outside the box” to problem-solve cases. In addition, participants believed that having an implementation process that was driven by a designated team that assisted with coordination, collaboration, coaching, and problem solving was helpful in SMHP implementation. Further, participants noted that having officers networked within the system of service providers was helpful in getting probationers connected to community supports and that SMHP activities gave them the opportunity to build these networks. Finally, SMHP implementation was aided by leaders within probation who were engaged in the planning process and who communicated their commitment to addressing mental illnesses among probationers.

Of the five CFIR domains, innovation characteristics was not indicated as a challenge or a facilitator. This is a notable omission given the complexity of the model itself. The authors anticipated that respondents would report challenges or frustration implementing a model that requires enhanced supervision and specialized training. However, the lack of respondents reporting challenges with the innovation itself should not be an indicator of a lack of challenges related to the model. Rather, the respondents may instead be focused on the challenges and facilitators related to how the model was implemented (i.e., implementation process) rather than the model itself (i.e., innovation characteristics). In addition, although characteristics of individuals emerged as a key facilitator, this domain was not named as a key challenge. The authors hypothesize that this omission is due both to a greater sense of self-efficacy among the respondents tasked with implementing the model as well as the reluctance of respondents to name challenges related to specific individuals involved in the pilot. Additional research is needed to determine whether the authors’ hypotheses are accurate.

### Implications

Although the implementation determinants described here were specific to a small pilot study, the results have a number of notable implications for SMHP implementation and other cross-sectoral interventions.

#### Assessing implementation determinants in a pilot study

A number of challenges emerged regarding the inner setting (i.e., probation) – specifically, workload capacity and resource availability issues – and the outer setting, such as connecting probationers to supports with an increasingly decentralized and diffuse mental health service delivery system. Such organizational and system-level challenges make it difficult to reach and maintain fidelity to cross-sectoral interventions, in general, and the SMHP model, in particular. These challenges may be further complicated when interventions aim to expand the roles of officers beyond the stated mission of the correctional agency (e.g., public safety).

With the complex and multi-component structure of SMHP and other types of cross-agency interventions, issues related to organizational context and the larger service system are context-dependent. For instance, interventions like SMHP that rely on connection to mental health services are dependent on the accessibility and availability of services in the local jurisdiction. However, accessibility and availability of services will vary across jurisdictions and will impact the implementation context differently. Further, the organizational context – e.g., vacancies, leadership engagement, commitment to intervention implementation – in which interventions are implemented are also likely to vary by jurisdiction. Consequently, the degree to which organizational factors are seen as challenges or facilitators will also vary.

Given the expected variation in the implementation context, criminal justice administrators should consider starting with a small pilot program and assess implementation determinants (Powell et al. 2015). A pilot study will provide critical information about potential implementation challenges and facilitators pertinent to the local context. Understanding the implementation determinants in the formative stages of an intervention can inform the larger implementation approach and provide guidance for a staged approach for scaling up the intervention (Colquhoun et al. 2017; Powell et al. 2015).

#### Focus on leadership engagement

In this pilot study, leadership and characteristics of top probation agency administrators were seen as essential assets during the implementation process. Given their role and influence within the organization, agency leaders were able to champion the value of SMHP in improving outcomes for individuals with mental illnesses. Having these leaders tie the purpose of such an intervention back to the agency’s primary mission of public safety and reducing recidivism could be a key strategy in building agency readiness prior to implementation (Weiner 2009). Efforts to engage these opinion leaders and change agents during the course of implementation could also help keep stakeholders on course when inevitable agency challenges arise.

Although many leaders referenced in this study were upper-level administrators, the value of leadership engagement among managers and supervisors should not be overlooked. Mid-level managers and supervisors provide essential information about the intervention, problem-solve challenges related to implementation, and bridge the gap between upper level management and those tasked with implementing the intervention on the front lines, thereby shaping the climate for implementation (Birken et al. 2012a, 2018). Within the context of SMHP, officers were directly supervised by the chief probation officer who staffed cases, controlled caseload size, and provided critical guidance on the use of sanctions. Agency administrators should consider the potential role of chief probation officers (as mid-level managers), engage these leaders in implementation planning, and encourage them to be pro-active problem-solvers during implementation efforts (Birken et al. 2012a, b).

#### Building inter-organizational networks

Successful implementation of a correctional health intervention like SMHP not only depends on the factors related to the host agency (i.e., inner setting) but also factors related to coordination and collaboration among entities within the external environment (i.e., outer setting). For example, in order for probation officers to successfully interface with local resources they should be informed about the types of services available, the specific agencies that provide services, and reliable methods for connecting with the agency. Consequently officer knowledge about, and relationships with, the existing resource network is paramount to the successful implementation of SMHP.

Agencies considering implementation of cross-agency interventions that rely on collaboration and coordination across multiple agencies and disciplines should focus on agency inter-relationships. Creating and enhancing stakeholder inter-relationships (Powell et al. 2015) may be a viable implementation strategy for agencies to consider in an overall implementation approach. For interventions like SMHP, agency administrators may consider creating an implementation strategy comprised of activities aimed at developing relationships across stakeholders (e.g., probation officers, mental health service providers, substance use service providers, housing providers). Such activities might include structured networking opportunities – e.g., service provider resource fairs – or enhancing activities that are already built into the role of the officers (e.g., attending a treatment team meeting). Also, workforce trainings show promise in other settings where front-line professionals’ familiarity with community-based services is important for promoting clients’ access to care in other systems (Fitzgerald et al. 2015). Developing the resource networks of officers has the potential to enhance implementation of SMHP and cross-agency correctional health interventions and improve outcomes.

### Limitations

There are a number of study limitations that warrant consideration. For example, subjectivity on the part of our qualitative data coders in the deductive coding process could have biased our findings in unknown ways. To minimize this limitation, we conducted consensus coding with a third member of our research team serving as an auditor to improve rigor and the trustworthiness of our results. In addition, our study lacks the perspectives of individuals on probation who received the intervention. Their firsthand perspectives are critical in understanding the implementation and the effectiveness of interventions at the nexus of corrections and mental health services. Moreover, unknown biases could have been introduced during the interviewing process given that some of the interviewers were part of the implementation team and also coded interviews. Again, a third member of our research team served as an auditor to improve rigor and the trustworthiness of our results. Finally, although our data are drawn from both rural and urban settings, the extent to which the findings from a small, local sample can be generalized to other criminal justice jurisdictions is unclear. Thus, this study should be replicated to further advance the literature.

## Conclusion

Specialty mental health probation is a complex and multi-component intervention that aims to improve criminal justice and mental health outcomes among people on probation who have serious mental illnesses. The complexity and cross-agency context of SMHP and other correctional health interventions introduces a number of challenges beyond the organizational context of the host agency. This study begins to address cross-agency implementation challenges and promotes the development of implementation strategies in this understudied area.

## Data Availability

Qualitative data analyzed in this study may be made available from the corresponding author upon reasonable request.
